# An efficient algorithm for testing the compatibility of phylogenies with nested taxa

**DOI:** 10.1186/s13015-017-0099-7

**Published:** 2017-03-16

**Authors:** Yun Deng, David Fernández-Baca

**Affiliations:** Department of Computer Science, Iowa State University, Atanasoff Hall, Ames, IA USA

**Keywords:** Algorithms, Phylogenetics, Supertrees, Taxonomies

## Abstract

**Background:**

Semi-labeled trees generalize ordinary phylogenetic trees, allowing internal nodes to be labeled by higher-order taxa. Taxonomies are examples of semi-labeled trees. Suppose we are given collection $\mathcal {P}$ of semi-labeled trees over various subsets of a set of taxa. The ancestral compatibility problem asks whether there is a semi-labeled tree that respects the clusterings and the ancestor/descendant relationships implied by the trees in $\mathcal {P}$. The running time and space usage of the best previous algorithm for testing ancestral compatibility depend on the degrees of the nodes in the trees in $\mathcal {P}$.

**Results:**

We give a algorithm for the ancestral compatibility problem that runs in $O(M_{\mathcal {P}}\log ^2 M_{\mathcal {P}})$ time and uses $O(M_{\mathcal {P}})$ space, where $M_{\mathcal {P}}$ is the total number of nodes and edges in the trees in $\mathcal {P}$.

**Conclusions:**

Taxonomies enable researchers to expand greatly the taxonomic coverage of their phylogenetic analyses. The running time of our method does not depend on the degrees of the nodes in the trees in $\mathcal {P}$. This characteristic is important when taxonomies—which can have nodes of high degree—are used.

## Introduction

In the *tree compatibility problem*, we are given a collection $\mathcal {P}= \{\mathcal {T}_1, \mathcal {T}_2, \ldots , \mathcal {T}_k\}$ of rooted phylogenetic trees with partially overlapping taxon sets. $\mathcal {P}$ is called a *profile* and the trees in $\mathcal {P}$ are the *input trees*. The question is whether there exists a tree $\mathcal {T}$ whose taxon set is the union of the taxon sets of the input trees, such that $\mathcal {T}$ exhibits the clusterings implied by the input trees. That is, if two taxa are together in a subtree of some input tree, then they must also be together in some subtree of $\mathcal {T}$. The tree compatibility problem has been studied for over three decades [1–4].

In the original version of the tree compatibility problem, only the leaves of the input trees are labeled. Here we study a generalization, called *ancestral compatibility*, in which taxa may be *nested*. That is, the internal nodes may also be labeled; these labels represent *higher-order taxa*, which are, in effect, sets of taxa. Thus, for example, an input tree may contain the taxon *Glycine max* (soybean) nested within a subtree whose root is labeled Fabaceae (the legumes), itself nested within an Angiosperm subtree. Note that leaves themselves may be labeled by higher-order taxa. The question now is whether there is a tree $\mathcal {T}$ whose taxon set is the union of the taxon sets of the input trees, such that $\mathcal {T}$ exhibits not only the clusterings among the taxa, but also the ancestor/descendant relationships among taxa in the input trees. Our main result is a $O(M_{\mathcal {P}}\log ^2 M_{\mathcal {P}})$ algorithm for the compatibility problem for trees with nested taxa, where $M_{\mathcal {P}}$ is the total number of nodes and edges in the trees in $\mathcal {P}$.

### Background

The tree compatibility problem is a basic special case of the *supertree problem*. A supertree method is a way to synthesize a collection of phylogenetic trees with partially overlapping taxon sets into a single supertree that represents the information in the input trees. The supertree approach, proposed in the early 90s [5, 6], has been used successfully to build large-scale phylogenies [7].

The original supertree methods were limited to input trees where only the leaves are labeled. Page [8] was among the first to note the need to handle phylogenies where internal nodes are labeled, and taxa are nested. A major motivation is the desire to incorporate *taxonomies* as input trees in large-scale supertree analyses, as way to circumvent one of the obstacles to building comprehensive phylogenies: the limited taxonomic overlap among different phylogenetic studies [9]. Taxonomies group organisms according to a system of taxonomic rank (e.g., family, genus, and species); two examples are the NCBI taxonomy [10] and the Angiosperm taxonomy [11]. Taxonomies spanning a broad range of taxa provide structure and completeness that might be hard to obtain otherwise. A recent example of the utility of taxonomies is the Open Tree of Life, a draft phylogeny for over 2.3 million species [12].

Taxonomies are not, strictly speaking, phylogenies. In particular, their internal nodes and some of their leaves are labeled with higher-order taxa. Nevertheless, taxonomies have many of the same mathematical characteristics as phylogenies. Indeed, both phylogenies and taxonomies are *semi-labeled trees* [13, 14]. We will use this term throughout the rest of the paper to refer to trees with nested taxa.

The fastest previous algorithm for testing ancestral compatibility, based on earlier work by Daniel and Semple [15], is due to Berry and Semple [16]. Their algorithm runs in $O \left( \log ^2 n \cdot \tau _\mathcal {P}\right)$ time using $O \left( \tau _\mathcal {P}\right)$ space. Here, *n* is the number of distinct taxa in $\mathcal {P}$ and $\tau _\mathcal {P}= \sum _{i = 1}^k \sum _{v \in I(\mathcal {T}_i)} d(v)^2$, where $I(\mathcal {T}_i)$ is the set of internal nodes of $\mathcal {T}_i$, for each $i \in \{1, \ldots , k\}$, and *d*(*v*) is the degree of node *v*. While the algorithm is polynomial, its dependence on node degrees is problematic: semi-labeled trees can be highly unresolved (i.e., contain nodes of high degree), especially if they are taxonomies.

### Our contributions

As stated earlier, our main result is an algorithm to test ancestral compatibility that runs in $O(M_{\mathcal {P}}\log ^2 M_{\mathcal {P}})$ time, using $O(M_{\mathcal {P}})$ space. These bounds are independent of the degrees of the nodes of the input trees, a valuable characteristic for large datasets that include taxonomies. To achieve our result, we extend ideas from our recent algorithm for testing the compatibility of ordinary phylogenetic trees [2]. As in that algorithm, a central notion in the current paper is the *display graph* of profile $\mathcal {P}$, denoted $H_{\mathcal {P}}$. This is the graph obtained from the disjoint union of the trees in $\mathcal {P}$ by identifying nodes that have the same label (see the section titled  "Testing ancestral compatibility"). The term “display graph” was introduced by Bryant and Lagergren [17], but similar ideas have been used elsewhere. In particular, the display graph is closely related to Berry and Semple’s *restricted descendancy graph* [16], a mixed graph whose directed edges correspond to the (undirected) edges of $H_{\mathcal {P}}$ and whose undirected edges have no correspondence in $H_{\mathcal {P}}$. The second kind of edges are the major component of the $\tau _\mathcal {P}$ term in the time and space complexity of Berry and Semple’s algorithm. The absence of such edges makes $H_{\mathcal {P}}$ significantly smaller than the restricted descendancy graph. Display graphs also bear some relation to *tree alignment graphs* [18].

Here, we exploit the display graph more extensively than in our previous work. Although the display graph of a collection of semi-labeled trees is more complex than that of a collection of ordinary phylogenies, we are able to extend several of the key ideas—notably, that of a semi-universal label—to the general setting of semi-labeled trees. As in [2], the implementation relies on a dynamic graph data structure, but it requires a more careful amortized analysis based on a weighing scheme.

### Contents

This paper has five sections, in addition to this introduction. The section titled "Preliminaries" presents basic definitions regarding graphs, semi-labeled trees, and ancestral compatibility. The section titled "The display graph" introduces the display graph and discusses its properties. The section titled "Testing ancestral compatibility" presents $\mathrm{BuildNT}$, our algorithm for testing ancestral compatibility. We first present the algorithm recursively, and then show how to transform it into an iterative algorithm, $\mathrm{BuildNT}_\mathrm{N}$, that is easier to implement. We also give an example of the execution of $\mathrm{BuildNT_N}$. The "Implementation" section gives the implementation details for $\mathrm{BuildNT_N}$. The "Discussion" section gives some concluding remarks.

## Preliminaries

For each positive integer *r*, [*r*] denotes the set $\{1, \ldots , r\}$.

### Graph notation

Let *G* be a graph. *V*(*G*) and *E*(*G*) denote the node and edge sets of *G*. The *degree* of a node $v \in V(G)$ is the number of edges incident on *v*. A *tree* is an acyclic connected graph. In this paper, all trees are assumed to be rooted. For a tree *T*, *r*(*T*) denotes the root of *T*. Suppose $u, v \in V(T)$. Then, *u* is an *ancestor* of *v* in *T*, denoted $u \le _T v$, if *u* lies on the path from *v* to *r*(*T*) in *T*. If $u \le _T v$, then *v* is a *descendant* of *u*. Node *u* is a *proper descendant* of *v* if *u* is a descendant of *v* and $v \ne u$. If $\{u,v\} \in E(T)$ and $u \le _T v$, then *u* is the *parent* of *v* and *v* is a *child* of *u*. If neither $u \le _T v$ nor $v \le _T u$ hold, then we write $u \parallel _T v$ and say that *u* and *v* are *not comparable* in *T*.

### Semi-labeled trees

A *semi-labeled tree* is a pair $\mathcal {T}= (T,\phi )$ where *T* is a tree and $\phi$ is a mapping from a set $L(\mathcal {T})$ to *V*(*T*) such that, for every node $v \in V(T)$ of degree at most two, $v \in \phi (L(\mathcal {T}))$. $L(\mathcal {T})$ is the *label set* of $\mathcal {T}$ and $\phi$ is the *labeling function* of $\mathcal {T}$.

For every node $v \in V(T)$, $\phi ^{-1}(v)$ denotes the (possibly empty) subset of $L(\mathcal {T})$ whose elements map into *v*; these elements as the *labels of* *v* (thus, each label is a taxon). If $\phi ^{-1}(v) \ne \emptyset$, then *v* is *labeled*; otherwise, *v* is *unlabeled*. Note that, by definition, every leaf in a semi-labeled tree is labeled. Further, any node, including the root, that has a single child must be labeled. Nodes with two or more children may be labeled or unlabeled. A semi-labeled tree $\mathcal {T}= (T,\phi )$ is *singularly labeled* if every node in *T* has at most one label; $\mathcal {T}$ is *fully labeled* if every node in *T* is labeled.

Semi-labeled trees, also known as *X* *-trees*, generalize ordinary phylogenetic trees, also known as *phylogenetic* *X* *-trees* [14]. An ordinary phylogenetic tree is a semi-labeled tree $\mathcal {T}= (T,\phi )$ where *r*(*T*) has degree at least two and $\phi$ is a bijection from $L(\mathcal {T})$ into leaf set of *T* (thus, internal nodes are not labeled).

Let $\mathcal {T}= (T,\phi )$ be a semi-labeled tree and let $\ell$ and $\ell '$ be two labels in $L(\mathcal {T})$. If $\phi (\ell ) \le _T \phi (\ell ')$, then we write $\ell \le _\mathcal {T}\ell '$, and say that $\ell '$ is a *descendant* of $\ell$ in $\mathcal {T}$ and that $\ell$ is an *ancestor* of $\ell '$. We write $\ell <_\mathcal {T}\ell '$ if $\phi (\ell ')$ is a proper descendant of $\phi (\ell )$. If $\phi (\ell ) \parallel _T \phi (\ell ')$, then we write $\ell \parallel _\mathcal {T}\ell '$ and say that $\ell$ and $\ell '$ are *not comparable* in $\mathcal {T}$. If $\mathcal {T}$ is fully labeled and $\phi (\ell )$ is the parent of $\phi (\ell ')$ in *T*, then $\ell$ is the *parent* of $\ell '$ in $\mathcal {T}$ and $\ell '$ is a *child* of $\ell$ in $\mathcal {T}$; two labels with the same parent are *siblings*.

Two semi-labelled trees $\mathcal {T}= (T,\phi )$ and $\mathcal {T}' = (T', \phi ')$ are *isomorphic* if there exists a bijection $\psi : V(T) \rightarrow V(T')$ such that $\phi ' = \psi \circ \phi$ and, for any two nodes $u, v \in V(T)$, $(u,v) \in E(T)$ if and only $(\psi (u), \psi (v)) \in E(T')$.

Let $\mathcal {T}= (T,\phi )$ be a semi-labeled tree. For each $u \in V(T)$, *X*(*u*) denotes the set of all labels in the subtree of *T* rooted at *u*; that is, $X(u) = \bigcup _{v: u \le _T v} \phi ^{-1}(v)$. *X*(*u*) is called a *cluster* of *T*. ${\mathrm{Cl}}(\mathcal {T})$ denotes the set of all clusters of $\mathcal {T}$. It is well known [14, Theorem 3.5.2] that a semi-labeled tree $\mathcal {T}$ is completely determined by ${\mathrm{Cl}}(\mathcal {T})$. That is, if ${\mathrm{Cl}}(\mathcal {T}) = {\mathrm{Cl}}(\mathcal {T}')$ for some other semi-labeled tree $\mathcal {T}'$, then $\mathcal {T}$ is isomorphic to $\mathcal {T}'$.

Suppose $A \subseteq L(\mathcal {T})$ for a semi-labeled tree $\mathcal {T}= (T,\phi )$. The *restriction* of $\mathcal {T}$ to *A*, denoted $\mathcal {T}|A$, is the semi-labeled tree whose cluster set is ${\mathrm{Cl}}(\mathcal {T}| A) = \{X \cap A : X \in {\mathrm{Cl}}(\mathcal {T}) \text { and } X \cap A \ne \emptyset \}.$ Intuitively, $\mathcal {T}| A$ is obtained from the minimal rooted subtree of *T* that connects the nodes in $\phi (A)$ by suppressing all vertices of degree two that are not in $\phi (A)$.

Let $\mathcal {T}= (T,\phi )$ and $\mathcal {T}' = (T', \phi ')$ be semi-labeled trees such that $L(\mathcal {T}') \subseteq L(\mathcal {T})$. $\mathcal {T}$ *ancestrally displays* $\mathcal {T}'$ if ${\mathrm{Cl}}(\mathcal {T}') \subseteq {\mathrm{Cl}}(\mathcal {T}|L(\mathcal {T}'))$. Equivalently, $\mathcal {T}$ ancestrally displays $\mathcal {T}'$ if $\mathcal {T}'$ can be obtained from $\mathcal {T}| L(\mathcal {T}')$ by contracting edges, and, for any $\ell _1, \ell _2 \in L(\mathcal {T}')$,

(i) if $\ell _1 <_\mathcal {\mathcal {T}'} \ell _2$, then $\ell _1 <_\mathcal {T}\ell _2$, and
(ii) if $\ell _1 \parallel _\mathcal {\mathcal {T}'} \ell _2$, then $\ell _1 \parallel _\mathcal {\mathcal {T}} \ell _2$.

The notion of “ancestrally displays” for semi-labeled trees generalizes the well-known notion of “displays” for ordinary phylogenetic trees [14].

For a semi-labelled tree $\mathcal {T}$, let us define $D(\mathcal {T})$ and $N(\mathcal {T})$ as follows.

$$\begin{aligned} D(\mathcal {T})&= \{(\ell ,\ell '): \ell , \ell ' \in L(\mathcal {T}) \text { and } \ell <_\mathcal {T}\ell '\} \\ N(\mathcal {T})&= \{\{\ell ,\ell '\} : \ell , \ell ' \in L(\mathcal {T}) \text { and } \ell \parallel _\mathcal {T}\ell '\} \end{aligned}$$

Note that $D(\mathcal {T})$ consists of *ordered* pairs, while $N(\mathcal {T})$ consists of *unordered* pairs.

#### **Lemma 1**

(Bordewich et al. [13])* Let* $\mathcal {T}$ *and* $\mathcal {T}'$ *be semi-labelled trees such that* $L(\mathcal {T}') \subseteq L(\mathcal {T})$.* Then* $\mathcal {T}$ *ancestrally displays* $\mathcal {T}'$ *if and only if* $D(\mathcal {T}') \subseteq D(\mathcal {T})$ *and* $N(\mathcal {T}') \subseteq N(\mathcal {T})$.

### Profiles and ancestral compatibility

**Fig. 1:**
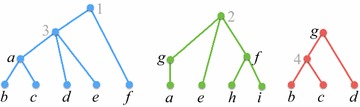
A profile $\mathcal {P}= \{\mathcal {T}_1, \mathcal {T}_2, \mathcal {T}_3\}$—trees are ordered left-to-right. The* letters* are the original labels;* grey numbers* are labels added to make the trees fully labeled (Adapted from [16])

Throughout the rest of this paper $\mathcal {P}= \{\mathcal {T}_1, \mathcal {T}_2, \ldots , \mathcal {T}_k\}$ denotes a set where, for each $i \in [k]$, $\mathcal {T}_i = (T_i, \phi _i)$ is a semi-labeled tree. We refer to $\mathcal {P}$ as a *profile*, and write $L(\mathcal {P})$ to denote $\bigcup _{i\in [k]} L(\mathcal {T}_i)$, the *label set* of $\mathcal {P}$. Figure 1 shows a profile where $L(\mathcal {P}) = \{a,b,c,d,e,f,g,h,i\}$. We write $V(\mathcal {P})$ for $\bigcup _{i\in [k]} V(T_i)$ and $E(\mathcal {P})$ for $\bigcup _{i\in [k]} E(T_i)$, The *size* of $\mathcal {P}$ is $M_{\mathcal {P}}= |V(\mathcal {P})| + |E(\mathcal {P})|$.

**Fig. 2:**
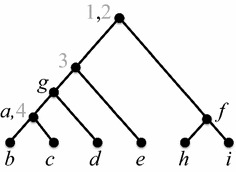
A tree $\mathcal {T}$ that ancestrally displays the profile of Fig. 1 (Adapted from [16])

$\mathcal {P}$ is *ancestrally compatible* if there is a rooted semi-labeled tree $\mathcal {T}$ that ancestrally displays each of the trees in $\mathcal {P}$. If $\mathcal {T}$ exists, we say that $\mathcal {T}$ *ancestrally displays* $\mathcal {P}$ (see Fig. 2).

Given a subset *X* of $L(\mathcal {P})$, the *restriction* of $\mathcal {P}$ to *X*, denoted $\mathcal {P}|X$, is the profile defined as

$$\begin{aligned} \mathcal {P}|X = \{\mathcal {T}_1|X \cap L(\mathcal {T}_1), \mathcal {T}_2|X \cap L(\mathcal {T}_2), \ldots , \mathcal {T}_k|X \cap L(\mathcal {T}_k)\}. \end{aligned}$$

The proof of the following lemma is straightforward.

#### **Lemma 2**

*Suppose* $\mathcal {P}$ *is ancestrally compatible and let* $\mathcal {T}$ *be a tree that ancestrally displays* $\mathcal {P}$.* Then, for any* $X \subseteq L(\mathcal {P})$, $\mathcal {T}| X$ *ancestrally displays* $\mathcal {P}| X$.

For technical reasons, fully labeled trees are easier to handle than those that are not. Suppose $\mathcal {P}$ contains trees that are not fully labeled. We can convert $\mathcal {P}$ into an equivalent profile $\mathcal {P}'$ of fully-labeled trees as follows. For each $i \in [k]$, let $l_i$ be the number of unlabeled nodes in $T_i$. Create a set $L'$ of $n' = \sum _{i \in [k]} l_i$ labels such that $L' \cap L(\mathcal {P}) = \emptyset$. For each $i \in [k]$ and each $v \in V(T_i)$ such that $\phi _i^{-1}(v) = \emptyset$, make $\phi _i^{-1}(v) = \{\ell \}$, where $\ell$ is a distinct element from $L'$. We refer to $\mathcal {P}'$ as the *profile obtained by adding distinct new labels to* $\mathcal {P}$ (see Fig. 1).

#### **Lemma 3**

(Daniel and Semple [15])* Let* $\mathcal {P}'$ *be the profile obtained by adding distinct new labels to* $\mathcal {P}$.* Then*, $\mathcal {P}$ *is ancestrally compatible if and only if* $\mathcal {P}'$ *is ancestrally compatible. Further, if* $\mathcal {T}$ *is a semi-labeled phylogenetic tree that ancestrally displays* $\mathcal {P}'$,* then* $\mathcal {T}$ *ancestrally displays* $\mathcal {P}$.

From this point forward, we make the following assumption.

#### **Assumption 1**

For each $i \in [k]$, $\mathcal {T}_i$ is fully and singularly labeled.

By Lemma 3, no generality is lost in assuming that all trees in $\mathcal {P}$ are fully labeled. The assumption that the trees are singularly labeled is inessential; it is only for clarity. Note that, even with the latter assumption, a tree that ancestrally displays $\mathcal {P}$ is not necessarily singularly labeled. Figure 2 illustrates this fact.

## The display graph

**Fig. 3:**
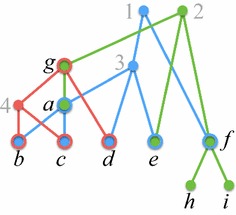
The display graph $H_{\mathcal {P}}$ for the profile of Fig. 1

The *display graph* of a profile $\mathcal {P}$, denoted $H_{\mathcal {P}}$, is the graph obtained from the disjoint union of the underlying trees $T_1, T_2, \ldots , T_k$ by identifying nodes that have the same label. Multiple edges between the same pair of nodes are replaced by a single edge. See Fig. 3.

$H_{\mathcal {P}}$ has $O(M_{\mathcal {P}})$ nodes and edges, and can be constructed in $O(M_{\mathcal {P}})$ time. By Assumption 1, there is a bijection between the labels in $L(\mathcal {P})$ and the nodes of $H_{\mathcal {P}}$. Thus, from this point forward, we refer to the nodes of $H_{\mathcal {P}}$ by their labels. It is easy to see that if $H_{\mathcal {P}}$ is not connected, then $\mathcal {P}$ decomposes into label-disjoint sub-profiles, and that $\mathcal {P}$ is compatible if and only if each sub-profile is compatible. Thus, without loss of generality, we shall assume the following.

### **Assumption 2**

$$H_{\mathcal {P}}$$ is connected.

### Positions

Our compatibility algorithm processes the trees in $\mathcal {P}$ from the top down, starting at the roots. We refer to the set of nodes in $\mathcal {P}$ currently being considered as a “position”. The algorithm advances from the current position to the next by replacing certain nodes in the current position by their children. Formally, a *position* (for $\mathcal {P}$) is a vector $U = (U(1), U(2), \ldots , U(k))$, where $U(i) \subseteq L(\mathcal {T}_i)$, for each $i \in [k]$. Since labels may be shared among trees, we may have $U(i) \cap U(j) \ne \emptyset$, for $i, j \in [k]$ with $i \ne j$. For each $i \in [k]$, let $\mathrm{Desc}_i(U) = \{\ell : \ell ' \le _{\mathcal {T}_i} \ell , \text { for some } \ell ' \in U(i)\}$, and let $\mathrm{Desc}_\mathcal {P}(U) = \bigcup _{i \in [k]} \mathrm{Desc}_i(U)$.

A position *U* is *valid* if, for each $i \in [k]$,

1. if $|U(i)| \ge 2$, then the elements of *U*(*i*) are siblings in $\mathcal {T}_i$ and
2. $$\mathrm{Desc}_i(U) = \mathrm{Desc}_\mathcal {P}(U) \cap L(\mathcal {T}_i)$$.

#### **Lemma 4**

*For any valid position* *U*,

$$\begin{aligned} \mathcal {P}| \mathrm{Desc}_\mathcal {P}(U) = \{\mathcal {T}_1|\mathrm{Desc}_1(U), \mathcal {T}_2|\mathrm{Desc}_1(U), \ldots , \mathcal {T}_k|\mathrm{Desc}_k(U)\}. \end{aligned}$$

#### *Proof*

By (V2), we have that $\mathcal {T}_i|\mathrm{Desc}_i(U)$ and $\mathcal {T}_i| \mathrm{Desc}_\mathcal {P}(U) \cap L(\mathcal {T}_i)$ are isomorphic, for each $i \in [k]$. The lemma then follows from the definition of $\mathcal {P}| \mathrm{Desc}_\mathcal {P}(U)$. $\square$

For any valid position *U*, $H_{\mathcal {P}}(U)$ denotes the subgraph of $H_{\mathcal {P}}$ induced by $\mathrm{Desc}_\mathcal {P}(U)$.

#### **Observation 1**

*For any valid position* *U*, $H_{\mathcal {P}}(U)$ *is the subgraph of* $H_{\mathcal {P}}$ *obtained by deleting all labels in* $V(H_{\mathcal {P}}) \setminus \mathrm{Desc}_\mathcal {P}(U)$,* along with all incident edges*.

A valid position of special interest to us is $U_\mathrm{root}$, the *root position*, defined as follows.

1 $$\begin{aligned} U_\mathrm{root}= (\phi _i^{-1}(r(T_1)), \phi _i^{-1}(r(T_2)), \ldots , \phi _i^{-1}(r(T_k))). \end{aligned}$$

That is, for each $i \in [k]$, $U_\mathrm{root}(i)$ is a singleton containing only the label of $r(T_i)$. In Fig. 3, $U_\mathrm{root}= (\{1\}, \{2\}, \{g\})$. It is straightforward to verify that $U_\mathrm{root}$ is indeed valid, that $\mathrm{Desc}_\mathcal {P}(U_\mathrm{root}) = L(\mathcal {P})$, and that $H_{\mathcal {P}}(U_\mathrm{root}) = H_{\mathcal {P}}$.

### Semi-universal labels

Let *U* be a valid position, and let $\ell$ be a label in *U*. Then, $\ell$ is *semi-universal in* *U* if $U(i) = \{\ell \}$, for every $i \in [k]$ such that $\ell \in L(\mathcal {T}_i)$. In Fig. 3, labels 1 and 2 are semi-universal in $U_\mathrm{root}$, but *g* is not, since *g* is in both $L(\mathcal {T}_2)$ and $L(\mathcal {T}_3)$, but $U_\mathrm{root}(2) \ne \{g\}$.

The term “semi-universal”, borrowed from Pe’er et al. [19], derives from the following fact. Suppose that $\mathcal {P}$ is ancestrally compatible, that $\mathcal {T}$ is a tree that ancestrally displays $\mathcal {P}$, and that $\ell$ is a semi-universal label for some valid position *U*. Then, as we shall see, $\ell$ must label the root $u_\ell$ of a subtree of $\mathcal {T}$ that contains all the descendants of $\ell$ in $\mathcal {T}_i$, for every *i* such that $\ell \in L(\mathcal {T}_i)$. The qualifier “semi” is because this subtree may also contain labels that do not descend from $\ell$ in any input tree, but descend instead from some other semi-universal label $\ell '$ in *U*. In this case, $\ell '$ also labels $u_\ell$. We exploit this property of semi-universal labels in our ancestral compatibility algorithm and its proof of correctness (see "Testing ancestral compatibility").

For each label $\ell \in L(\mathcal {P})$, let $k_\ell$ denote the number of input trees that contain label $\ell$. We can obtain $k_\ell$ for every $\ell \in L(\mathcal {P})$ in $O(M_{\mathcal {P}})$ time during the construction of $H_{\mathcal {P}}$.

#### **Lemma 5**

*Let* $U = (U(1), \ldots , U(k))$ *be a valid position*.* Then, label* $\ell$ *is semi-universal in* *U* *if the cardinality of the set* $J_\ell = \{i \in [k] : U(i) = \{\ell \}\}$ *equals* $k_\ell$.

#### *Proof*

By definition, $U(i) = \{\ell \}$, for every $i \in J_\ell$. Since $|J_\ell | = k_\ell$, the lemma follows. $\square$

### Successor positions

For every $i \in [k]$ and every $\ell \in L(\mathcal {T}_i)$, let ${\mathrm{Ch}}_i(\ell )$ denote the set of children of $\ell$ in $L(\mathcal {T}_i)$. For a subset *A* of $L(\mathcal {T}_i)$, let ${\mathrm{Ch}}_i(A) = \bigcup _{\ell \in A} {\mathrm{Ch}}_i(\ell )$. Let *U* be a valid position, and *S* be the set of semi-universal labels in *U*. The *successor of* *U* *with respect to* *S* is the position $U' = (U'(1), U'(2), \ldots , U'(k))$, where, for each $i \in [k]$, $U'(i)$ is defined as follows.

$$\begin{aligned} U'(i) = {\left\{ \begin{array}{ll} {\mathrm{Ch}}_i(\ell ) &{}\text {if } U(i) = \{\ell \}, \text { for some } \ell \in S, \\ U(i) &{} \text {otherwise.} \end{array}\right. } \end{aligned}$$

In Fig. 3, the set of semi-universal labels in $U_\mathrm{root}$ is $S = \{1, 2\}$. Since ${\mathrm{Ch}}_1(1) = \{3,f\}$ and ${\mathrm{Ch}}_2(2) = \{e,f,g\}$, the successor of $U_\mathrm{root}$ is $U_\mathrm{root}' = (\{3,f\}, \{e,f,g\}, \{g\})$.

#### **Observation 2**

*Let U be a valid position, and let* $U'$ *be the successor of* *U* *with respect to the set* *S* *of semi-universal labels in* *U*.* Then*, $H_{\mathcal {P}}(U')$ *can be obtained from* $H_{\mathcal {P}}(U)$ *by doing the following for each* $\ell \in S$:* (1) for each* $i \in [k]$ *such that* $U(i) = \{\ell \}$,* delete all edges between* $\ell$ *and* ${\mathrm{Ch}}_i(\ell )$;* (2) delete* $\ell$.

Let *U* be a valid position, and *W* be a subset of $\mathrm{Desc}_\mathcal {P}(U)$. Then, *U*|*W* denotes the position $(U(1) \cap W, U(2) \cap W, \ldots , U(k) \cap W)$. In Fig. 3, the components of $H_{\mathcal {P}}(U')$, where $U'$ is the successor of $U_\mathrm{root}$, are $W_1 = \{3,4,a,b,c,d,e,g\}$ and $W_2 = \{f,h,i\}$. Thus, $U' | W_1 = (\{3\}, \{e,g\}, \{g\})$ and $U' | W_2 = (\{f\}, \{f\}, \emptyset )$. We have the following result.

#### **Lemma 6**

*Let* *U* *be a valid position, and* *S* *be the set of all semi-universal labels in* *U*.* Let* $U'$ *be the successor of* *U* *with respect to* *S*,* and let* $W_1, W_2, \ldots , W_p$ *be the label sets of the connected components of* $H_{\mathcal {P}}(U')$.* Then,* $U' | W_j$ *is a valid position, for each* $j \in [p]$.

#### *Proof*

It suffices to argue that $U'$ satisfies conditions (V1) and (V2). The lemma then follows from the fact that the connected components of $H_{\mathcal {P}}(U')$ are label-disjoint.

$U'$ must satisfy condition (V1), since *U* does. Suppose $\ell \in S$. Then, for each $i \in [k]$ such that $\ell \in L(\mathcal {T}_i)$, $\mathrm{Desc}_i(U') = \mathrm{Desc}_i(U) \setminus \{\ell \}$ and $\mathrm{Desc}_\mathcal {P}(U') \cap L(\mathcal {T}_i) =(\mathrm{Desc}_\mathcal {P}(U) \cap L(\mathcal {T}_i)) \setminus \{\ell \}$. Thus, since (V2) holds for *U*, it also holds for $U'$. $\square$

## Testing ancestral compatibility

### Overview of the algorithm

$\mathrm{BuildNT}$ (Algorithm 1) is our algorithm for testing compatibility of semi-labeled trees. Its argument, *U*, is a valid position in $\mathcal {P}$ such that $H_{\mathcal {P}}(U)$ is connected. $\mathrm{BuildNT}$ relies on the fact—proved later, in Theorem 1—that if $\mathcal {P}|\mathrm{Desc}_\mathcal {P}(U)$ is compatible, then *U* must contain a nonempty set *S* of semi-universal labels. If such a set *S* exists, the algorithm replaces *U* by its successor $U'$ with respect to *S*. It then processes each connected component of $H_{\mathcal {P}}(U')$ recursively, to determine if the associated sub-profile is compatible. If all the recursive calls are successful, then their results are combined into a supertree for $\mathcal {P}|\mathrm{Desc}_\mathcal {P}(U)$.

In detail, $\mathrm{BuildNT}$ proceeds as follows. Line 1 computes the set *S* of semi-universal labels in *U*. If *S* is empty, then, $\mathcal {P}|\mathrm{Desc}_\mathcal {P}(U)$ is incompatible, and, thus, so is $\mathcal {P}$. This fact is reported in Line 3. Line 4 creates a tentative root $r_U$, labeled by *S*, for the tree $\mathcal {T}_U$ for *L*(*U*). Line 5 checks if *S* contains exactly one label $\ell$, with no proper descendants. If so, by the connectivity assumption, $\ell$ must be the sole member of $\mathrm{Desc}_\mathcal {P}(U)$; that is, $L(U) = \ell$. Therefore, Line 6 simply returns the tree with a single node, labeled by $S = \{\ell \}$. Line 7 updates *U*, replacing it by its successor with respect to *S*. Let $W_1, W_2, \dots , W_p$ be the connected components of $H_{\mathcal {P}}(U)$ after updating *U*. By Lemma 6, $U | W_j$ is a valid position, for each $j \in [p]$. Lines 8–12 recursively invoke $\mathrm{BuildNT}$ on $U | W_j$ for each $j \in [p]$, to determine if there is a tree $t_j$ that ancestrally displays $\mathcal {P}| \mathrm{Desc}_\mathcal {P}(U \cap W_j)$. If any subproblem is incompatible, Line 12 reports that $\mathcal {P}$ is incompatible. Otherwise, Line 13 returns the tree obtained by making the $t_j$s the subtrees of root $r_U$. 
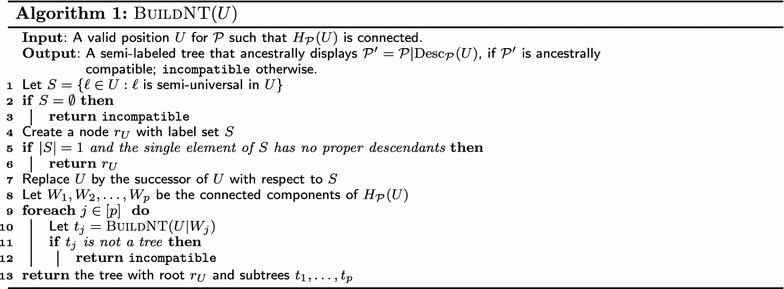


Next, we argue the correctness of $\mathrm{BuildNT}$.

### Correctness

#### **Lemma 7**

*Let* *U* *be a valid position in* $\mathcal {P}$.* If* $\mathrm{BuildNT}(U)$ *returns a tree* $\mathcal {T}_U$,* then* $\mathcal {T}_U$ *is a phylogenetic tree such that* $L(\mathcal {T}_U) = L(U)$.

#### *Proof*

We use induction on |*L*(*U*)|. The base case, where $|L(U)| = 1$, is handled by Lines 5–6. In this case, $S = L(U) = \{\ell \}$ and $\mathrm{BuildNT}(U)$ correctly returns the tree consisting of a single node, labeled by $\{\ell \}$. Otherwise, let $W_1, \ldots , W_p$ be the connected components of $H_{\mathcal {P}}(U)$ in step 8. Since $\mathrm{BuildNT}(U)$ returns tree $\mathcal {T}_U$, it must be the case that, for each $j \in [p]$, the result $t_j$ returned by the recursive call to $\mathrm{BuildNT}(U|W_j)$ in Line 10 is a tree. Since $|S| \ge 1$, we have $|L(W_j)| < |L(U)|$, for each $j \in [p]$. Thus, we can assume inductively that $t_j$ is a phylogenetic tree for $L(W_j)$. Since $S \cup \bigcup _{j \in [p]} L(W_j) = L(U)$, the tree returned in Line 13 is a phylogeny with species set *L*(*U*). $\square$

#### **Theorem 1**

*Let* $\mathcal {P}= \{\mathcal {T}_1, \mathcal {T}_2, \ldots , \mathcal {T}_k\}$ *be a profile and let* $U_\mathrm{root}$ *be the root position, as defined in Eq. *(1). * Then,* $\mathrm{BuildNT}(U_\mathrm{root})$ *returns either (i) a semi-labeled tree* $\mathcal {T}$ *that ancestrally displays* $\mathcal {P}$,* if* $\mathcal {P}$ *is ancestrally compatible, or (ii)* incompatible *otherwise*.

#### *Proof*

$\mathrm{BuildNT}(U_\mathrm{root})$ either returns a tree or incompatible. We consider each case separately.

(i) Suppose that $\mathrm {BuildNT}(U_\mathrm{root})$ returns a semi-labeled tree $\mathcal {T}$. By Lemma 7, $L(\mathcal {T}) = L(\mathcal {P})$. We prove that $\mathcal {T}$ ancestrally displays $\mathcal {P}$. By Lemma 1, it suffices to show that $D(\mathcal {T}_i) \subseteq D(\mathcal {T})$ and $N(\mathcal {T}_i) \subseteq N(\mathcal {T})$, for each $i \in [k]$. Consider any $(\ell ,\ell ') \in D(\mathcal {T}_i)$. Then, $\ell$ has a child $\ell ''$ in $\mathcal {T}_i$ such that $\ell '' \le _{\mathcal {T}_i} \ell '$ —note that we may have $\ell '' = \ell$. There must be a recursive call to $\mathrm {BuildNT}(U)$, for some valid position *U*, where $\ell$ is the set *S* of semi-universal labels obtained in Line 1. By Observation 2, label $\ell ''$, and thus $\ell '$, both lie in one of the connected components of the graph obtained by deleting all labels in *S*, including $\ell$, and their incident edges from $H_{\mathcal {P}}(U)$. It now follows from the construction of $\mathcal {T}$ that $(\ell , \ell ') \in D(\mathcal {T})$. Thus, $D(\mathcal {T}_i) \subseteq D(\mathcal {T})$. Now, consider any $\{\ell ,\ell ' \} \in N(\mathcal {T}_i)$. Let *v* be the lowest common ancestor of $\phi _i(\ell )$ and $\phi _i(\ell ')$ in $\mathcal {T}_i$ and let $\ell _v$ be the label of *v*. Then, $\ell _v$ has a pair of children, $\ell _1$ and $\ell _2$ say, in $\mathcal {T}_i$ such that $\ell _1 \le _{\mathcal {T}_i} \ell$, and $\ell _2 \le _{\mathcal {T}_i} \ell '$. Because $\mathrm {BuildNT}(U_\mathrm{root})$ returns a tree, there are recursive calls $\mathrm {BuildNT}(U_1)$ and $\mathrm {BuildNT}(U_2)$ for valid positions $U_1$ and $U_2$ such that $\ell _1$ is semi-universal for $U_1$ and $\ell _2$ is semi-universal for $U_2$. We must have $U_1 \ne U_2$; otherwise, $|U_1(i)| = |U_2(i)| \ge 2$, and, thus, neither $\ell _1$ nor $\ell _2$ is semi-universal, a contradiction. Further, it follows from the construction of $\mathcal {T}$ that we must have $\mathrm{Desc}_\mathcal {P}(U_1) \cap \mathrm{Desc}_\mathcal {P}(U_2) = \emptyset$. Hence, $\ell \parallel _\mathcal {T}\ell '$, and, therefore, $\{\ell ,\ell '\} \in N(\mathcal {T})$.
(ii) Asssume, by way of contradiction, that $\mathrm{BuildNT}(U_\mathrm{root})$ returns *incompatible*, but that $\mathcal {P}$ is ancestrally compatible. By assumption, there exists a semi-labeled tree $\mathcal {T}$ that ancestrally displays $\mathcal {P}$. Since $\mathrm {BuildNT}(U_\mathrm{root})$ returns *incompatible*, there is a recursive call to $\mathrm{BuildNT}(U)$ for some valid position *U* such that *U* has no semi-universal label, and the set *S* of Line 1 is empty. By Lemma 2, $\mathcal {T}| \mathrm{Desc}_\mathcal {P}(U)$ ancestrally displays $\mathcal {P}|\mathrm{Desc}_\mathcal {P}(U)$. Thus, by Lemma 4, $\mathcal {T}| \mathrm{Desc}_\mathcal {P}(U)$ ancestrally displays $\mathcal {T}_i | \mathrm{Desc}_i(U)$, for every $i \in [k]$. Let $\ell$ be any label in the label set of the root of $\mathcal {T}| \mathrm{Desc}_\mathcal {P}(U)$. Then, for each $i \in [k]$ such that $\ell \in L(\mathcal {T}_{i})$, $\ell$ must be the label of the root of $\mathcal {T}_i|\mathrm{Desc}_i(U)$. Thus, for each such *i*, $U(i) = \{\ell \}$. Hence, $\ell$ is semi-universal in *U*, a contradiction.

$$\square$$

### An iterative version

We now present $\mathrm {BuildNT}_\mathrm {N}$ (Algorithm 2), an iterative version of $\mathrm {BuildNT}$, which lends itself naturally to an efficient implementation. $\mathrm{BuildNT_N}$ performs a breadth-first traversal of $\mathrm{BuildNT}$’s recursion tree, using a first-in first-out queue *Q* that stores pairs of the form $(U, \mathrm{pred})$, where *U* is a valid position in $\mathcal {P}$ and $\mathrm{pred}$ is a reference to the parent of the node corresponding to *U* in the supertree built so far. $\mathrm{BuildNT_N}$ simulates recursive calls in $\mathrm{BuildNT}$ by enqueuing pairs corresponding to subproblems. We explain this in more detail next.

$\mathrm {BuildNT_N}$ initializes its queue to contain the starting position, $U_\mathrm{root}$, with a null parent. It then proceeds to the* while* loop of Lines 3–14. Each iteration of the loop starts by dequeuing a valid position *U*, along with a reference $\mathrm{pred}$ to the potential parent for the subtree for *L*(*U*) in the supertree. The body of the loop closely follows the steps performed by a call to $\mathrm{BuildNT}(U)$. Line 5 computes the set *S* of semi-universal labels in *U*. If *S* is empty, the algorithm reports that $\mathcal {P}$ is incompatible and terminates (Lines 6–7). The algorithm then creates a tentative root $r_U$ labeled by *S* for the tree $\mathcal {T}_U$ for *L*(*U*), and links $r_U$ to its parent (Line 8). If *S* consists of exactly one element that has no proper descendants, we skip the rest of the current iteration of the* while* loop, and* continue* to the next iteration (Lines 9–10). Line 11 replaces *U* by its successor with respect to *S*. Lines 13–14 enqueue each of $U|W_1, U|W_2, \ldots , U|W_p$, along with $r_U$, for processing in a subsequent iteration. If the* while* loop terminates without any incompatibility being detected, the algorithm returns the tree with root $r_{U_\mathrm{root}}$. 
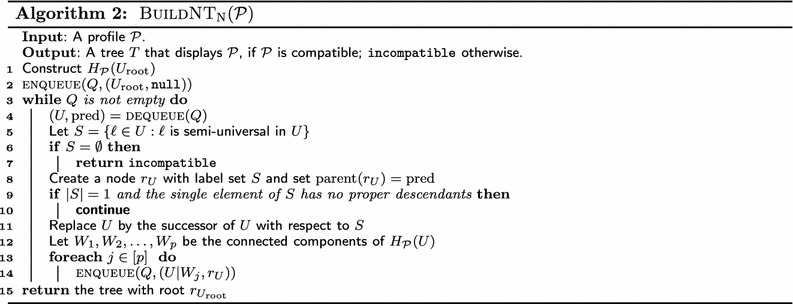


Although the order in which $\mathrm{BuildNT_N}$ processes connected components differs from that of $\mathrm {BuildNT}$ —breadth-first instead of depth-first—, it is straightforward to see that the effect is equivalent, and the proof of correctness of $\mathrm{BuildNT}$ (Theorem 1) applies to $\mathrm{BuildNT_N}$ as well. We thus state the following without proof.

#### **Theorem 2**

*Let* $\mathcal {P}= \{\mathcal {T}_1, \mathcal {T}_2, \ldots , \mathcal {T}_k\}$ *be a profile. Then,* $\mathrm{BuildNT_N}(\mathcal {P})$ *returns either (i) a semi-labeled tree* $\mathcal {T}$ *that ancestrally displays* $\mathcal {P}$,* if* $\mathcal {P}$ i*s ancestrally compatible, or (ii)* incompatible *otherwise*.

Let *Q* be $\mathrm{BuildNT_N}$’s first-in first-out queue. In the rest of the paper, we will say that a valid position *U* *is in* *Q* if $(U,\mathrm{pred}) \in Q, \text { for some } \mathrm{pred}.$ Let $H_Q$ be the subgraph of $H_{\mathcal {P}}$ induced by $\bigcup \{\mathrm{Desc}(U): U \text { is in } Q\}.$ By Observation 1, $H_Q$ is obtained from $H_{\mathcal {P}}$ through edge and node deletions.

#### **Lemma 8**

*At the start of any iteration of* $\mathrm{BuildNT_N}$’*s* *while loop, the set of connected components of* $H_Q$ *is* $\{V(H_{\mathcal {P}}(U)) : U \text { is in } Q\}$.

#### *Proof*

The property holds at the outset, since, by Assumption 2, $H_{\mathcal {P}}= H_{\mathcal {P}}(U_\mathrm{root})$ is a connected graph, and the only element of *Q* is $(U_\mathrm{root},\mathtt {null})$. Assume that the property holds at the beginning of iteration *l*. Let $(U, \mathrm{pred})$ be the element dequeued from *Q* in Line 4. Then, $H_{\mathcal {P}}(U)$ is connected. In place of $(U, \mathrm{pred})$, Lines 13–14 enqueue $(U|W_j, r_U)$, for each $j \in [p]$, where, by construction, $H_{\mathcal {P}}(U|W_j)$ is a connected component of $H_{\mathcal {P}}(U)$. Thus, the property holds at the beginning of iteration $l+1$. $\square$

In other words, Lemma 8 states that each iteration of $\mathrm{BuildNT_N}(\mathcal {P})$ deals with a subgraph of $H_{\mathcal {P}}$, whose connected components are in one-to-one correspondence with the valid positions stored in *Q*. This is illustrated by the next example.

### An example

**Fig. 4:**
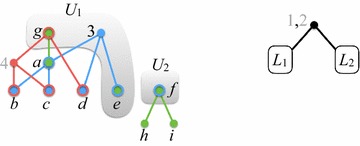
After generating all supertree nodes in level 0

**Fig. 5:**
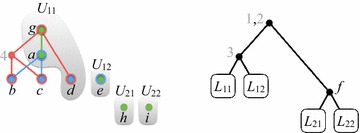
After generating all supertree nodes in level 1

**Fig. 6:**
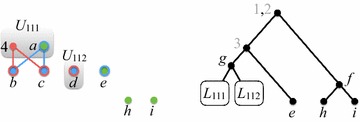
After generating all supertree nodes in level 2

**Fig. 7:**
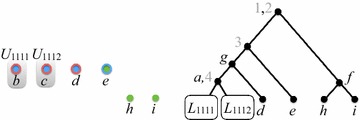
After generating all supertree nodes in level 3

**Fig. 8:**
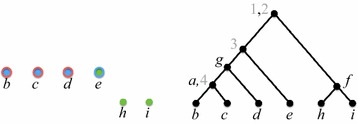
After generating all supertree nodes in level 4

Figures 4, 5, 6, 7 and 8 illustrate the execution of $\mathrm{BuildNT_N}$ on the profile $\mathcal {P}= (\mathcal {T}_1, \mathcal {T}_2, \mathcal {T}_3)$ of Fig. 1. The figures show how the graph $H_Q$ —initially equal to $H_{\mathcal {P}}= H_{\mathcal {P}}(U_\mathrm{root})$ (Fig. 3)—evolves as its edges and nodes are deleted.

In each figure, $H_Q$ is shown on the left and the current supertree is shown on the right. For brevity, the figures only exhibit the state of $H_Q$ and the supertree after all the nodes at each level are generated. The various valid positions processed by $\mathrm{BuildNT_N}(\mathcal {P})$ are denoted by $U_\alpha$, for different subscripts $\alpha$; $S_\alpha$ denotes the semi-universal labels in $U_\alpha$, and $U_\alpha '$ denotes the successor of $U_\alpha$ with respect to $S_\alpha$. We write $L_\alpha$ as an abbreviation for $L(U_\alpha )$ The root of the tree for $L_\alpha$ is $r_{U_\alpha }$ and is labeled by $S_\alpha$.

Initially, $Q = ((U_\mathrm{root},\mathtt {null}))$. In what follows, the elements of *Q* are listed from front to rear.

*Level 0.* Refer to Fig. 4. As seen earlier, the set of semi-universal labels of $U_\mathrm{root}$ is $S_\mathrm{root}= \{1, 2\}$. Thus, $H_{\mathcal {P}}(U_\mathrm{root}')$ has two components $W_1$ and $W_2$. Let $U_1 = U_\mathrm{root}'|W_1$ and $U_2 = U_\mathrm{root}'|W_2$. Then,

$$\begin{aligned} U_1 = (\{3\}, \{e,g\},\{g\}) \quad \text {and} \quad U_2 = (\{f\}, \{f\},\emptyset ). \end{aligned}$$

After level 0 is processed, $Q = ((U_1,r_{U_\mathrm{root}}), (U_2,r_{U_\mathrm{root}}))$. Thus, the roots of the subtrees for $L_1$ and $L_2$ will be children of $r_{U_\mathrm{root}}$.

*Level 1.* Refer to Fig. 5. We have $S_1 = \{3\}$, so $H_{\mathcal {P}}(U_1')$ has two components $W_{11}$ and $W_{12}$. Let $U_{11} = U_1'|W_{11}$ and $U_{12} = U_1'|W_{12}$. Then,

$$\begin{aligned} U_{11} = (\{a,d\}, \{g\},\{g\}) \quad \text {and} \quad U_{12} = (\{e\}, \{e\},\emptyset ). \end{aligned}$$

We have $S_2 = \{f\}$, so $H_{\mathcal {P}}(U_2')$ has two components $W_{21}$ and $W_{22}$. Let $U_{21} = U_2'|W_{21}$ and $U_{22} = U_2'|W_{22}$. Then,

$$\begin{aligned} U_{21} = (\emptyset , \{h\},\emptyset ) \quad \text {and} \quad U_{21} = (\emptyset , \{i\},\emptyset ). \end{aligned}$$

After level 1 is processed, $Q = ((U_{11},r_{1}), (U_{12},r_{1}), (U_{21},r_{2}), (U_{22},r_{2}))$.

*Level 2.* Refer to Fig. 6. We have $S_{11} = \{g\}$, so $H_{\mathcal {P}}(U_{11}')$ has two components $W_{111}$ and $W_{112}$. Let $U_{111} = U_{11}'|W_{111}$ and $U_{112} = U_{11}'|W_{112}$. Then,

$$\begin{aligned} U_{111} = (\{a\}, \{a\},\{4\}) \quad \text {and} \quad U_{112} = (\emptyset , \{d\},\{d\}). \end{aligned}$$

The only semi-universal labels in $U_{12}$, $U_{21}$, and $U_{22}$ are, respectively, *e*, *h*, and *i*. Since none of these labels have proper descendants, each of them is a leaf in the supertree.

After level 2 is processed, $Q = ((U_{111},r_{11}), (U_{112},r_{11}))$.

*Level 3.* Refer to Fig. 7. We have $S_{111} = \{4, a\}$, so $H_{\mathcal {P}}(U_{111}')$ has two components $W_{1111}$ and $W_{1112}$. Let $U_{1111} = U_{111}'|W_{1111}$ and $U_{1112} = U_{111}'|W_{1112}$. Then,

$$\begin{aligned} U_{1111} = (\{b\}, \emptyset ,\{b\}) \quad \text {and} \quad U_{1112} = (\{c\}, \emptyset ,\{c\}). \end{aligned}$$

The only semi-universal label in $U_{112}$ is *d*. Since *d* has no proper descendants, it becomes a leaf in the supertree.

After level 3 is processed, $Q = ((U_{1111},r_{111}), (U_{1112},r_{111}))$.

*Level 4.* Refer to Fig. 8. The only semi-universal labels in $U_{1111}$ and $U_{1112}$ are, respectively, *b* and *c*. Since neither of these labels have proper descendants, each of them is a leaf in the supertree.

After level 4 is processed, *Q* is empty, and $\mathrm{BuildNT_N}(\mathcal {P})$ terminates.

## Implementation

Here we prove the following result.

### **Theorem 3**

*There is an algorithm that, given a profile* $\mathcal {P}$ *of rooted trees, runs in* $O(M_{\mathcal {P}}\log ^2 M_{\mathcal {P}})$ *time, and either returns a tree that displays* $\mathcal {P}$,* if* $\mathcal {P}$ *is compatible, or reports that is* $\mathcal {P}$ *is incompatible otherwise*.

We prove this theorem by showing how to implement $\mathrm{BuildNT_N}$ so that the algorithm runs in $O(M_{\mathcal {P}}\log ^2 M_{\mathcal {P}})$ on any profile $\mathcal {P}$.

As in the section titled "An iterative version", let $H_Q$ denote the subgraph of $H_{\mathcal {P}}$ associated with the valid positions in $\mathrm{BuildNT_N}$’s queue. By Lemma 8, each valid position *U* in *Q* corresponds to one connected component of $H_Q$ —namely $\mathrm{Desc}(U)$ —and vice-versa. We use this fact in the implementation of $\mathrm{BuildNT_N}$: alongside each valid position *U* in *Q*, we also store a reference to the respective connected component, together with additional information, described next, to quickly identify semi-universal labels.

Let *U* be any valid set in *Q*, let $Y = V(H_{\mathcal {P}}(U))$ be the corresponding connected component of $H_Q$, and let $\ell$ be any label in *Y*. Our implementation maintains the following data fields.

- Let $J_U = \{i \in [k] : U(i) \ne \emptyset \}$. Then, $Y.\mathtt {map}$ is a map from $J_U$ to *L*(*U*), where, for each $i \in J_U$, $Y.\mathtt {map}(i) = U(i)$.
- For each $\ell \in Y$, $\ell .\mathtt {count}$ equals the cardinality of the set $\{i \in [k] : Y.\mathtt {map}(i) = \{\ell \}\}$. (Recall that $k_\ell$ is the number of input trees that contain $\ell$.).
- $Y.\mathtt {exposed}$, a set consisting of all $i \in [k]$ such that $Y.\mathtt {map}(i) = \{\ell \}$ for some $\ell \in Y$ such that $\ell .\mathtt {count}= k_\ell$.
- $Y.\mathtt {weight}$, which equals $\sum _{\ell \in Y} k_\ell$. This field is needed for technical reasons, to be explained later.

For the purpose of analysis, we assume that the $\mathtt {exposed}$ fields are represented as balanced binary search trees (BSTs), which ensures $O(\log k) = O(\log M_{\mathcal {P}})$ time per access and update. The $\mathtt {map}$ fields are also implemented using BSTs. We store the set $J_U = \{i \in [k] : U(i) \ne \emptyset \}$ as a BST, enabling is to determine in $O(\log k)$ time if an index *i* is in $J_U$, and, if this is the case, to access $Y.\mathtt {map}(i)$. The latter is also stored as a BST, allowing us to search and update $Y.\mathtt {map}(i)$ in $O(\log |U(i)|) = O(\log M_{\mathcal {P}})$ time. Note that, in practice, hashing may be a better alternative for both $\mathtt {exposed}$ and $\mathtt {map}$ fields, as it offers expected constant time performance per operation.

The data fields listed above allow us to efficiently retrieve the set *S* of semi-universal labels in *U*, as needed in line 5 of $\mathrm{BuildNT_N}(\mathcal {P})$. Indeed, suppose that *U* is the valid position extracted from *Q* at the beginning of an iteration of the* while* loop of Lines 3–14, and that $Y = V(H_{\mathcal {P}}(U))$. Then, by Lemma 5, we have $S = \{v \in Y.\mathtt {map}(i) : i \in Y.\mathtt {exposed}\}$. What remains is to devise an efficient way to update these fields for each of the connected components of $H_{\mathcal {P}}(U)$ created by replacing *U* with its successor in Line 11.

Let $U'$ be the value of *U* after Line 11; thus, $U'$ is the successor of *U*. By Observation 2, $H_{\mathcal {P}}(U')$ is obtained from $H_{\mathcal {P}}(U)$ through edge and node deletions. We need to

- Generate the new connected components resulting from these deletions, and
- Produce the required $\mathtt {map}$, $\mathtt {count}$, and $\mathtt {exposed}$ data fields for the various connected components.

We accomplish (a) using the dynamic graph connectivity data structure of Holm et al. [20], which we refer to as *HDT*. HDT allows us to maintain the list of nodes in each component, as well as the number of these nodes so that, if we start with no edges in a graph with *N* nodes, the amortized cost of each update is $O(\log ^2 N)$. Since $H_{\mathcal {P}}$ has $O(M_{\mathcal {P}})$ nodes, each update takes $O(\log ^2 M_{\mathcal {P}})$ time. The total number of edge and node deletions performed by $\mathrm{BuildNT_N}(\mathcal {P})$ —including all deletions in the interations—is at most the total number of edges and nodes in $H_{\mathcal {P}}$, which is $O(M_{\mathcal {P}})$. HDT allows us to maintain connectivity information throughout the entire algorithm in $O(M_{\mathcal {P}}\log ^2 M_{\mathcal {P}})$ time, which is within the time bound claimed in Theorem 3.

For part (b), we need to augment HDT in order to maintain the the required data fields for the various connected components created during edge and node deletion. In the next subsections, we describe how to do this. We begin by explaining how to initialize all the required data fields for $H_{\mathcal {P}}= H_{\mathcal {P}}(U_\mathrm{root})$.

### Initializing the data fields

Graph $H_{\mathcal {P}}(U_\mathrm{root})$ has a single connected component, $Y_\mathrm{root}= L(\mathcal {P})$, which is the entire vertex set of the graph. We initialize the data fields as follows.

- For each $i \in [k]$, $Y_\mathrm{root}.\mathtt {map}(i) = \{ r(T_i)\}$. This takes *O*(*k*) time.
- $Y_\mathrm{root}.\mathtt {weight}= \sum _{\ell \in L(\mathcal {P})} k_\ell$. This takes $O(M_{\mathcal {P}})$ time.

We initialize the $\mathtt {count}$ fields in $O(M_{\mathcal {P}})$ time as follows:

1. Set $\ell .\mathtt {count}$ to 0 for all $\ell \in L(\mathcal {P})$.
2. For each $i \in [k]$, do the following.
  - Let $\rho _i$ denote the label of $r(T_i)$.
  - Increment $\rho _i.\mathtt {count}$ by one.

Once the $\mathtt {count}$ fields are initialized, it is easy to initialize $Y_\mathrm{root}.\mathtt {exposed}$ in *O*(*k*) time. Thus, we can initialize all the required fields in $O(M_{\mathcal {P}})$ time.

### Maintaining the data fields

Suppose that all data fields fields are correctly computed for every connected component that is in *Q* at the beginning of an iteration of the* while* loop in 3–14 of $\mathrm{BuildNT_N}$. We now show how to generate the same fields efficiently for the new connected components created by Line 11.

#### Computing successor positions

Let *U* be the valid position extracted from *Q* at the beginning of an iteration of $\mathrm{BuildNT_N}$’s* while* loop, and let $Y = V(\mathrm{Desc}(U))$ be the associated connected component. Assume all the data fields for *Y* have been correctly computed. To obtain the successor of *U* in Line 11 of $\mathrm{BuildNT_N}$, we perform the following steps.

1. Identify the set *S* of semi-universal labels in *U*. As we saw, this set is given by $S = \{\ell \in Y.\mathtt {map}(i) : i \in Y.\mathtt {exposed}\}$.
2. Set $Y.\mathtt {map}(i) = \emptyset$, for every $i \in Y.\mathtt {exposed}$.
3. Make $$Y.\mathtt {exposed}= \emptyset$$.
4. For each $\ell \in S$ and each *i* such that $\ell \in L(T_i)$, do the following.
  If ${\mathrm{Ch}}_i(\ell ) \ne \emptyset$, replace $Y.\mathtt {map}(i)$ by ${\mathrm{Ch}}_i(\ell )$. If ${\mathrm{Ch}}_i(\ell )$ is a singleton set $\{\alpha \}$, increment $\alpha .\mathtt {count}$ by one. If $\alpha .\mathtt {count}= k_\ell$, add *i* to $Y.\mathtt {exposed}$.
  Otherwise, $Y.\mathtt {map}(i)$ is undefined.
5. For each label $\ell$ in *S*, delete the edges incident on $\ell$ and then $\ell$ itself, updating the data fields as necessary after each deletion.

The total number of operations on $\mathtt {map}$ and $\mathtt {exposed}$ fields in Steps 1–4 is $O(\sum _{\ell \in S} k_\ell )$. Since each label becomes semi-universal at most once, the total number of operations on $\mathtt {map}$ fields over the entire execution of $\mathrm{BuildNT_N}(\mathcal {P})$ is $O(\sum _{\ell \in L(\mathcal {P})} k_\ell )$, which is $O(M_{\mathcal {P}})$. The same bound holds for updates to $\mathtt {count}$ and $\mathtt {exposed}$ fields.

Next let us focus on how to handle the deletion of a single edge in Step 5.

#### Deleting an edge

To delete an edge between $\ell$ and a child $\alpha$ of $\ell$, we proceed as follows.

1. Delete $(\ell ,\alpha )$, querying HDT to determine whether this disconnects *Y*.
  If *Y* remains connected, skip the next steps and proceed directly to the next child of $\ell$.
  Otherwise, *Y* is split into two components, $Y_1$ and $Y_2$.
2. Update $$Y_1.\mathtt {weight}$$ and $$Y_2.\mathtt {weight}$$.
3. Identify which of $Y_1$ and $Y_2$ has the smaller $\mathtt {weight}$ field. Without loss of generality, assume that $Y_1.\mathtt {weight}\le Y_2.\mathtt {weight}$.
4. Initialize $Y_1.\mathtt {map}$ and $Y_1.\mathtt {exposed}$ to $\mathtt {null}$.
5. Initialize $Y_2.\mathtt {map}$ and $Y_2.\mathtt {exposed}$ to $Y.\mathtt {map}$ and $Y.\mathtt {exposed}$, respectively.
6. For each label $\beta$ in $Y_1$, perform the following steps for each *i* such that $\beta \in L(\mathcal {T}_i)$.
  - Delete $\beta$ from $Y_2.\mathtt {map}(i)$ and add $\beta$ to $Y_1.\mathtt {map}(i)$.
  - Adjust $\mathtt {count}$ and $\mathtt {exposed}$ fields as necessary.

The connectivity test in Step 1 is done by querying HDT. Steps 3–5 are trivial. We thus focus on Steps 2 and 6.

To perform Step 2, we use the well-known technique of scanning the smaller component [21]. We first consult HDT to determine which of $Y_1$ or $Y_2$ has fewer nodes. Assume, without loss of generality, that $|Y_1| \le |Y_2|$. We initialize $Y_1.\mathtt {weight}$ to 0 and $Y_2.\mathtt {weight}$ to $Y.\mathtt {weight}$. We then scan the labels of $Y_1$, incrementing $Y_1.\mathtt {weight}$ by $k_\ell$ for each label $\ell \in Y_1$. When the scan of $Y_1$ is complete, we make $Y_2.\mathtt {weight}= Y_2.\mathtt {weight}- Y_1.\mathtt {weight}$. We claim that any label $\ell \in L(\mathcal {P})$ is scanned $O(\log M_{\mathcal {P}})$ times over the entire execution of $\mathrm{BuildNT_N}(\mathcal {P})$. To verify this, let $N(\ell )$ be the number of nodes in the connected component containing $\ell$. Suppose that, initially, $N(\ell ) = N$. Then, the *r*th time we scan $\ell$, $N(\ell ) \le N/2^r$. Thus, $\ell$ is scanned $O(\log N)$ times. The claim follows, since $N = O(M_{\mathcal {P}})$. Therefore, the total number of updates over all labels is $O(M_{\mathcal {P}}\log M_{\mathcal {P}})$.

Each execution of Step 6(a) updates each of $Y_1.\mathtt {map}(i)$ and $Y_2.\mathtt {map}(i)$ once. Step 6(b) is more complex, but can also be accomplished with *O*(1) data field updates. We omit the (tedious) details. In summary, each execution of step 6 for some $\beta \in L(\mathcal {P})$ performs $O(k_\beta )$ data field updates.

Let us track the number of data field updates in Step 6 that can be attributed to some specific label $\beta \in L(P)$ over the entire execution of $\mathrm{BuildNT_N}(\mathcal {P})$. Let $w_r(\beta )$ be the weight of the connected component containing $\beta$ at the beginning of Step 6, on the *r*th time that $\beta$ is considered in that step. Thus, $w_0(\beta ) \le \sum _{\ell \in L(\mathcal {P})} k_\ell$. We claim that $w_r(\beta ) \le w_0(\beta )/2^r$. The reason is that we only consider $\beta$ if (a) $\beta$ is contained in one of the two components that result from deleting an edge in step 1 and (b) the component containing $\beta$ has the smaller weight. Hence, the number of times $\beta$ is considered in step 6 over the entire execution of $\mathrm{BuildNT_N}(\mathcal {P})$ is $O(\log w_0(\beta ))$, which is $O(\log M_{\mathcal {P}})$, since $w_0(\beta ) = O(M_{\mathcal {P}})$. Therefore, the total number of data field updates in Step 6, over all labels in $L(\mathcal {P})$ considered throughout the entire execution of $\mathrm{BuildNT_N}(\mathcal {P})$, is $O(\log M_{\mathcal {P}}\cdot \sum _{\ell \in L(\mathcal {P})} k_\ell )$, which is $O(M_{\mathcal {P}}\log M_{\mathcal {P}})$.

### Summary

Let us review the running times of each aspect of our implementation of $\mathrm{BuildNT_N}$.

- *Initializing the data structures*. This has two parts.
  - *Setting up the HDT data structure for* $H_{\mathcal {P}}$. This takes $O(M_{\mathcal {P}}\log ^2 M_{\mathcal {P}})$ time.
  - *Initializing the data fields for the single connected component of* $H_{\mathcal {P}}$. This takes $O(M_{\mathcal {P}})$ time.
- *Maintaining the data structures*. This also has two parts.
  - *Updating the HDT data structure*. There are $O(M_{\mathcal {P}})$ edge and node deletions, at an amortized cost of $O(\log ^2 M_{\mathcal {P}})$ per deletion, yielding a total time of $O(M_{\mathcal {P}}\log ^2 M_{\mathcal {P}})$.
  - *Maintaining the relevant data fields for the connected components.* We have seen that the total number of updates is $O(M_{\mathcal {P}}\log M_{\mathcal {P}})$. Assume, conservatively, that each update can be done in $O(\log M_{\mathcal {P}})$ time. Then, this part takes a total of $O(M_{\mathcal {P}}\log ^2 M_{\mathcal {P}})$ over the entire execution of $\mathrm{BuildNT_N}$.

We conclude that the total running time of $\mathrm{BuildNT_N}(\mathcal {P})$ is $O(M_{\mathcal {P}}\log ^2 M_{\mathcal {P}})$, completing the proof of Theorem 3.

## Discussion

Like our earlier algorithm for compatibility of ordinary phylogenetic trees, the more general algorithm presented here, $\mathrm{BuildNT_N}$, is a polylogarithmic factor away from optimality (a trivial lower bound is $\Omega (M_{\mathcal {P}})$, the time to read the input). $\mathrm{BuildNT_N}$ has a linear-space implementation, using the results of Thorup [22]. A question to be investigated next is the performance of the algorithm on real data. Another important issue is integrating our algorithm into a synthesis method that deals with incompatible profiles.
